# Supplementary material for: Brain-derived estrogens facilitate male-typical behaviors by potentiating androgen receptor signaling in medaka
Source: eLife. 2026 Jan 13;13:RP97106. doi: 10.7554/eLife.97106 (PMC12799210; doi:10.7554/eLife.97106)
Supplement: Supplementary file 1. [file elife-97106-supp1.pdf]

Supplementary file 1. Abbreviations of brain nuclei.

abbreviation	full name	location
aNVT	anterior part of NVT	hypothalamus
aPMp	anterior part of PMp	preoptic area
aPPp	anterior part of PPp	preoptic area
NAT	anterior tuberal nucleus	hypothalamus
NPT	posterior tuberal nucleus	hypothalamus
NRL	lateral recess nucleus	hypothalamus
NVT	ventral tuberal nucleus	hypothalamus
PMg	gigantocellular portion of the magnocellular preoptic nucleus	preoptic area
PMm	magnocellular portion of the magnocellular preoptic nucleus	preoptic area
PMp	parvocellular portion of the magnocellular preoptic nucleus	preoptic area
pNVT	posterior part of NVT	hypothalamus
PPa	anterior parvocellular preoptic nucleus	preoptic area
pPMp	posterior part of PMp	preoptic area
PPp	posterior parvocellular preoptic nucleus	preoptic area
pPPp	posterior part of PPp	preoptic area
SC	suprachiasmatic nucleus	preoptic area
VM	ventromedial nucleus	thalamus
Vp	posterior nucleus of the ventral telencephalic area	ventral telencephalon
Vs	supracommissural nucleus of the ventral telencephalic area	ventral telencephalon
Vv	ventral nucleus of the ventral telencephalic area	ventral telencephalon
