# Supplementary material for: Brain-derived estrogens facilitate male-typical behaviors by potentiating androgen receptor signaling in medaka
Source: eLife. 2026 Jan 13;13:RP97106. doi: 10.7554/eLife.97106 (PMC12799210; doi:10.7554/eLife.97106)
Supplement: Supplementary file 2. [file elife-97106-supp2.pdf]

Supplementary file 2. Primers and probes used in this study.

primer/ probe	target	direction	purpose	sequence (5′ to 3′)
primer	*cyp19a1b*	forward	genotyping (gDNA PCR)	GACTTGGTCCTGTCCTGTCCTA
primer	*cyp19a1b*	reverse	genotyping (gDNA PCR)	ATCCTGGTTTTCTTCCACAGAG
primer	*cyp19a1b*	forward	genotyping (CS)	TTGTGAGGGTATGGATTAATGG
primer	*cyp19a1b*	forward	genotyping (HRM)	CGGCTGAAAGCTTGTTTACCTA
primer	*cyp19a1b*	reverse	genotyping (HRM)	CCCGAATCTAGACGTGTAGTGG
probe	*cyp19a1b*	forward	genotyping (HRM)	TGAGGTGTACCATGTTTTGAAGAGC
primer	*cyp19a1b*	forward	real-time PCR	AAGAAGATGATCCAGCAAGAG
primer	*cyp19a1b*	reverse	real-time PCR	AGCATCAGAAGAAGTAAGAAAAGTG
primer	*esr2a*	forward	genotyping (gDNA PCR)	ATGTCGCTTTTGCAGTTTAAGCTG
primer	*esr2a*	reverse	genotyping (gDNA PCR)	ATGAACACGGATCTGCTGATGG
primer	*esr2a*	forward	genotyping (CS)	ACGGCTTTGAAGATCCTTGGCT
primer	*esr2a*	forward	genotyping (HRM)	CAGGCGGCAAGTCTGAACTC
primer	*esr2a*	reverse	genotyping (HRM)	CTCCATTTTACCTTGGATGCTCC
probe	*esr2a*	forward	genotyping (HRM)	ATACCACTACGGCGTGTGGTCATGCGAG

gDNA PCR, PCR on genomic DNA; CS, cycle sequence; HRM, high-resolution melting analysis.
